# Microsatellite Polymorphism in the Heme Oxygenase-1 Gene Promoter and the Risk of Atrial Fibrillation in Taiwanese

**DOI:** 10.1371/journal.pone.0108773

**Published:** 2014-09-30

**Authors:** Lung-An Hsu, Yung-Hsin Yeh, Chi-Tai Kuo, Ying-Hwa Chen, Gwo-Jyh Chang, Feng-Chun Tsai, Wei-Jan Chen

**Affiliations:** 1 Cardiovascular Division, Chang Gung Memorial Hospital, Chang Gung University College of Medicine, Tao-Yuan, Taiwan; 2 Division of Cardiology, Department of Internal Medicine, Taipei Veterans General Hospital, National Yang-Ming University College of Medicine, Taipei, Taiwan; 3 Graduate Institute of Clinical Medical Sciences, Chang Gung University, Tao-Yuan, Taiwan; 4 Division of Cardiac Surgery, Chang Gung Memorial Hospital, Chang Gung University College of Medicine, Tao-Yuan, Taiwan; University of Louisville, United States of America

## Abstract

**Background:**

Atrial fibrillation (AF) is associated with increased oxidative stress. Emerging evidence suggests that heme oxygenase-1 (HO-1) is a potent antioxidant system against various oxidative stress-related diseases. The human HO-1 promoter has a GT-repeat length polymorphism that can determine the level of gene transcription.

**Objective:**

The aim of this study is to assess the role of the GT-repeat polymorphism in the promoter region of the HO-1 gene in Chinese-Taiwanese patients with AF.

**Methods and Results:**

This study enrolled 200 AF patients and 240 controls, comparable for age and gender. In each subject, the length of GT-repeat polymorphism in the HO-1 promoter region was examined by polymerase chain reactions. The frequencies of long GT-repeat alleles (≧32) were significantly higher in AF patients than in controls. Multivariate analysis showed that the presence of long allele was significantly and independently associated with AF (odds ratio: 1.91, 95% CI 1.07–3.72; *P* = 0.028). Right atrial tissues from patients with chronic AF were investigated with immunoconfocal microscopy. Patients *homozygous* for shorter GT-repeat alleles exhibited greater HO-1 expression in their atria than those *homozygous* for longer alleles, which was reflected by less oxidative stress, myofibril degradation, and fibrosis in the atria of patients with shorter GT-repeat. *In vitro*, transient transfection assay in HL-1 atrial myocytes showed that the responsiveness of HO-1 transcriptional activity to tachypacing was inversely correlated with the length of the GT-repeats.

**Conclusion:**

Our results suggest that the HO-1 microsatellite polymorphism may contribute to the genetic background of AF in Chinese-Taiwanese patients.

## Introduction

Atrial fibrillation (AF), an abnormality of heart rhythm, occurs as a consequence of atrial electrical or structural remodeling [1]. AF is frequently associated with other cardiovascular diseases, such as hypertension, ischemic heart disease, valvular heart disease, and heart failure. However, some AF patients (10–20%) are free of concomitant diseases and explainable causes (so-called lone AF) [1].

Although AF is originally thought to be a non-genetic disorder, emerging evidence has pointed to a heritable linkage in many patients [2], [3]. Population-based studies have demonstrated a significant contribution of single-gene mutations and single-nucleotide polymorphisms in the development of AF. For instance, variations or polymorphisms in ion channels, calcium handling proteins, fibrosis, conduction, and inflammation-related genes may render distinct individuals susceptible to electrical or structural remodeling and eventually fibrillation in the atrium [2], [3].

Heme oxygenase-1 (HO-1), a stress-responsive protein that can be induced by various oxidative conditions, degrades heme into 3 products: Fe^2+^, biliverdin, and CO [4]. The inducible HO-1 also acts as antioxidant system and provides protection against a variety of oxidative stress-related diseases [4], [5]. However, the responsiveness of HO-1 varies among individuals with different inherited settings. The length polymorphism of (GT)n dinucleotide repeats in the human HO-1 promoter may determine the transcriptional activity of HO-1 gene [6], [7]. Individuals with longer GT-repeat lengths exhibit not only lower HO-1 expression but also decreased antioxidant capacities against oxidative stress [6]–[8]. The involvement of HO-1 microsatellite polymorphisms in many cardiovascular diseases, such as coronary artery disease (CAD) and restenosis after stenting, has been subjected to intense investigation [7], [9]–[11]. Nevertheless, relatively little is known about its role in predisposing AF. Since many studies have linked oxidative stress to the pathogenesis of AF [12]–[15], the aim of this study is to assess whether the microsatellite polymorphism in the *HO-1* gene promoter is associated with AF in the Chinese-Taiwanese population.

## Materials and Methods

### Ethics Statement

The protocols were approved by the Human Research Ethics Committee at Chang Gung Memorial Hospital (Chang Gung Medical Foundation Institutional Review Board 100–1901B and 100–3196C1) and were conducted in concordance with the Declaration of Helsinki Principles. Written informed consent was obtained from each subject.

### Study population

The study enrolled patients who were less than 65 years old and had unexplained causes of AF. Patients who had a history of hyperthyroidism, significant valvular heart disease (>grade II mitral regurgitation and/or aortic regurgitation), or congestive heart failure (left ventricular ejection fraction <50%) were excluded. The control group with sinus rhythm (SR), comparable for age and gender, was recruited from a population receiving routine health examinations. The demographic details of the AF patients and control subjects have been described elsewhere [16].

### Clinical assessment

The presence of AF was documented by patient history, serial electrocardiograms (ECGs), and/or ambulatory ECG monitoring. Transthoracic echocardiography was performed to assess left atrial and left ventricular functions and to detect significant valvular diseases. Left atrial enlargement and left ventricular dysfunction were defined as diameter >40 mm and ejection fraction <50%, respectively. Hypertension was diagnosed as ≥140/90 mmHg and/or the use of antihypertensive medication. Definitions of hypercholesterolemia and diabetes mellitus were in accordance with the third report of the National Cholesterol Education Program and the guidelines of the American Diabetes Association, respectively.

### Genomic DNA extraction

Genomic DNA was extracted from peripheral blood leukocytes and/or tissues using the Puregene DNA Isolation Kit (Qiagen, Minneapolis, MN).

### Genotyping of the HO-1 promoter microsatellite polymorphism

The 5′-flanking region of the HO-1 gene containing GT-repeats was amplified by polymerase chain reaction (PCR) with a FAM-labeled sense primer (5′-AGAGCCTGCAGCTTCTCAGA-3′) and an antisense primer (5′-ACAAAGTCTGGCCATAGGAC-3′) according to a published protocol [7]. The PCR products were mixed with the GenoType TAMRA DNA Ladder (size range: 50–500 bp; Invitrogen, Grand Island, NY) and analyzed in an automated DNA sequencer (ABI Prism 377, Foster City, CA). The respective sizes of the GT-repeats were calculated using GeneScan Analysis software (PE Applied Biosystems, Foster City, CA). To further confirm the sizes of the GT-repeats, 3 PCR products were subcloned into the pCRII vector (Invitrogen) and subjected to the sequence analysis. For quality control purposes, approximately 10% of the samples were re-genotyped in a blinded fashion, and from which the same results were obtained.

### Human samples

Right atrial appendages were obtained from 34 patients with AF and 4 controls with SR undergoing open-heart surgery. After excision, atrial appendages were immediately frozen in liquid nitrogen and stored at −85°C. Subsequently, genomic DNA from each subject was sent for genotyping as described above.

### Immunohistochemical analysis

Immunohistochemical analysis was performed using α-actin, HO-1, myosin heavy chain (MHC), and collagen I primary antibodies (Abcam, Cambridge, MA) followed by fluorescein isothiocyanate (FITC) or Cy3-conjugated secondary antibodies (Chemicon, Temecula, CA). Nuclei were visualized by DAPI-staining. The expression levels of target proteins were calculated as protein-occupied area in the tissue divided by the nuclear area. Myosin degradation was quantified as cytoplasmic myosin (MHC)-area divided by the nuclear area. For each analysis, at least 5 random fields were chosen to observe >30 myocytes. Atrial fibrosis was quantified by collagen deposition as previously described [17], [18]. Reactive oxygen species (ROS) in the atria were measured using a fluorescent dye dihydroethidium (DHE), a cell-permeable ROS indicator. Tissues were pre-incubated with 10 µmol/L DHE for 30 minutes at room temperature. The ROS-mediated fluorescence was observed under a confocal microscope (Leica TCS SP2, Wetzlar, Germany) with excitation at 543 nm argon laser and emission was recorded using a longpass LP>600 nm filter set to acquire two-dimensional images (512×512 pixel).

### Cell culture and tachypacing

HL-1 atrial myocytes were maintained in Claycomb medium as described previously [19]. HL-1 cells (≥1×10^6^ cells) on 4-well rectangular dishes (Nunclon, Netherlands) were placed into C-Dish 100TM-Culture Dishes (IonOptix, Milton, MA). HL-1 cells were then subjected to field stimulation with 10-ms stimuli of 40-V intensity at 4 Hz frequency (1.5-V/cm field strength; C-Pace EP culture pacer, IonOptix) [20]–[23]. The spontaneous contraction rate was about 0.5–1 Hz and capture efficiency of >90% was confirmed by microscopic examination.

### Constructs and transfection

The HO-1 promoters (−4.9 kb to +0.3 kb) containing various lengths of GT-repeats were amplified from genomic DNAs of AF patients by PCR using the forward primer 5′-TGCGTATGTGTGTGTGTATTGC-3′ and an reverse primer 5′-CTGAGGACGCTCGAGAGGAG-3′ according to a published protocol [24]. The PCR products were inserted into the pGL3-basic vector (Promega, Madison, WI) at the *Sac*I/*Xho*I restriction sites. For transient transfection assays, HL-1 myocytes grown to 50–60% confluence were transfected with the indicated plasmids using LipofectAMINE 2000 (Invitrogen) according to the manufacturer’s instructions. The transfection efficiency by this method was approximately 60%. Co-transfection of a β-galactosidase expression vector was served as an internal control for normalizing the transfection efficiency. Luciferase activities were measured with a luminometer (Luminoskan TL PMS, Thermo Lab Systems, Grand Rapids, OH).

### Statistical analysis

Continuous variables were expressed as mean ± SEM and tested using a two-sample *t-*test. The chi-square test was used to examine the differences in categorical variables and to compare the allele and genotype frequencies. Binary logistic regression analysis was used to evaluate the independent effect of genotype on the association with AF after adjustment for age, gender, body mass index (BMI), hypertension, diabetes, smoking, hypercholesterolemia, CAD, and concomitant medication use. Unpaired Student’s *t*-test and one-way ANOVA with post hoc Tukey’s tests were applied for 2 groups and multiple comparisons, respectively. A value of *p*<0.05 using a two-sided test was considered statistically significant. All statistical analyses were performed using SPSS software, version 20.0 (SPSS Inc, Chicago, IL).

## Results

### GT-repeat length polymorphism in the HO-1 promoter is associated with AF

Table 1 presents the baseline characteristics of 200 AF patients and 240 controls. The frequencies of classical risk factors for AF, such as hypertension, diabetes, and history of CAD, were slightly, but not significantly, higher in AF patients than in controls. Concomitant medications were significantly different between these 2 groups. The allele frequencies of the GT-repeats in our study population were shown in Figure S1. The GT-repeat numbers in controls ranged from 14 to 40 with (GT)_23_ and (GT)_30_ being the 2 most common alleles, which is consistent with those reported in a Taiwanese population [7]. Therefore, we classified these alleles into 3 subgroups in accordance with the best-fit grouping criteria, as confirmed by a sensitivity analysis: short alleles (class S), <23 repeats; median alleles (class M), between 23 and 32 repeats; long alleles (class L), ≧32 repeats. The overall distribution of allele frequencies was significantly different between AF patients and controls (*P* = 0.016; Table 2). The proportions of allelic frequencies in class L and class S were higher (*P* = 0.024) and lower (*P* = 0.037), respectively, in AF patients than in controls. With respect to genotype distribution, AF patients also carried higher frequencies of L allele-containing genotypes (L/L, L/M, and L/S) than controls (28.0% versus 18.8%; *P* = 0.022; Table 2). L-allele carriers had a greater risk of AF than did the non-L-allele carriers (M/M, M/S, and S/S) (odds ratio (OR): 1.69; 95% CI = 1.08 to 2.64). On multivariate analysis, the presence of L allele was significantly and independently associated with the risk of AF after adjustment for age, gender, BMI, hypertension, diabetes, smoking, hypercholesterolemia, CAD, and concomitant medication use (OR: 1.91, 95% CI = 1.07–3.72; *P* = 0.028).

**Table 1:** Demographic and Clinical Characteristics of the Study Population.

	Controls	AF patients	P
	(n = 240)	(n = 200)	
Age, years	55.7±7.6	56.9±8.4	0.14
Gender (M/F)	172/68	145/55	0.85
BMI, kg/m^2^	25.3±3.2	25.5±4.5	0.68
Hypertension, n (%)	126 (52.5)	119 (59.5)	0.14
Diabetes mellitus, n (%)	17 (7.1)	21 (10.5)	0.20
Smoking, n (%)	62 (25.8)	45 (22.6)	0.43
Hypercholesterolemia, n (%)	25 (10.4)	21 (10.5)	0.98
CAD, n (%)	5 (2.1)	10 (5.0)	0.09
paroxysmal/persistent, n (%)	–	109/91 (54.5/45.5)	
LA dimension >40 mm, n (%)	–	86 (43.0)	
ARB, n (%)	65 (27.1)	83 (41.5)	<0.001
ACE inhibitor, n (%)	15 (6.2)	11 (5.5)	0.74
β-blocker, n (%)	56 (23.3)	88 (44.0)	<0.001
Calcium antagonist, n (%)	71 (29.6)	79 (39.5)	0.03
Diuretic, n (%)	11 (4.6)	32 (16.0)	<0.001
Digoxin, n (%)	0 (0.0)	34 (17.0)	<0.001
Statin, n (%)	41 (17.1)	59 (29.5)	0.002
Aspirin, n (%)	17 (7.1)	85 (42.5)	<0.001
Oral anticoagulant, n (%)	0 (0.0)	38 (19.0)	<0.001
Systemic embolization, n (%)	0 (0.0)	9 (4.5)	0.001

ACE = angiotensin converting enzyme; ARB = angiotensin receptor blocker;

BMI = body mass index; CAD = coronary artery disease; LA = left atrium.

**Table 2:** Genotype distribution and allele frequencies of HO-1 promoter in the study population.

	Control	AF	P
	(n = 240)	(n = 200)	
Alleles, n (%)			0.016
S (n<23)	69 (14.4%)	39 (9.8% )	
M (23≦n<32)	364 (75.8%)	302 (75.5%)	
L (n≧32)	47 (9.8%)	59 (14.7%)	
Genotypes, n (%)			0.09
S/S	6 (2.5%)	4 (2.0%)	
M/S	50 (20.8% )	24 (12.0% )	
L/S	7 (2.9%)	7 (3.5%)	
M/M	139 (57.9%)	116 (58.0% )	
L/M	36 (15.0%)	46 (23.0%)	
L/L	2 (0.8%)	3 (1.5%)	
L-allele carrier	45 (18.8%)	56 (28.0%)	0.022
Non-L-allele carrier	195 (81.2%)	144 (72.0%)	

### Association of GT-repeat length polymorphism with HO-1 expression in atrial tissues

**Figure 1:**
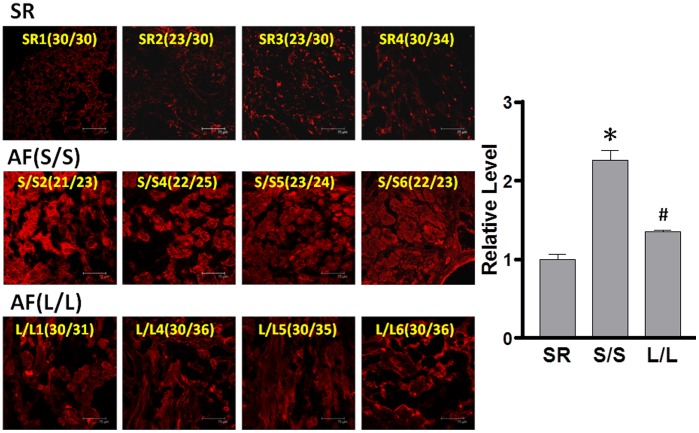
Association of GT-repeat length polymorphism with HO-1 expression in atrial tissues. Representative confocal images show the expression of HO-1 in the atria of 8 AF patients and 4 controls (sinus rhythm subjects [SR], left). The number represents the size of GT-repeats in each subject. Relative intensity of HO-1 measured in the α-actin-expressing areas was quantified (right). S/S denotes 4 of 6 patients with shorter GT-repeat (<27 GT) homozygous genotype and L/L denotes 4 of 6 patients with longer (≧27 GT) homozygous genotype. At least 5 random fields were chosen to observe >30 myocytes with scanning and averaging. Data are expressed as mean ± SE. P<0.05; *, #: the different symbols represent the significant difference among groups.

A recent report has shown higher HO-1 levels in the atria of AF patients than those of SR subjects [25]. Accordingly, the next experiments were designed to assess whether the GT-repeat length polymorphism is associated with the HO-1 expression in AF tissues. Of the 34 AF patients, 6 were homozygous for short repeat alleles (<27 GT repeat) and 6 were homozygous for long repeat alleles (≧27 GT repeat). These 12 samples were chosen for comparison. Four SR patients were designated as controls. Table 3 displays the baseline characteristics of these 16 patients. The HO-1 expression in myocytes (identified by co-localization with α-actin) was greater in the atria of AF patients homozygous for short repeat alleles than that homozygous for long repeat and SR controls, although AF patients homozygous for long repeat alleles still had higher HO-1 levels than SR controls (Figure 1). These data implicate that the increased HO-1 levels in AF patients may depend on their inherited backgrounds.

**Table 3:** Clinical characteristics of patients with normal SR and AF at the time of cardiac surgery.

				Underlying cardiac disease								Previous medical history					
No.	Age (yr)	Sex	GT-repeatnumber	CAD	OperativeIndication	Duration ofAF (yr)	DM		hypertension	LV EjectionFraction (%)	LAD (mm)	β blocker	Digitalis	statins	Diuretics	ACE inhibitorsor ARB	Calcium channelBlockers
**SR**																	
1	79	M	30/30	+	CABG	−	−		+	65	31	+	−	−	+	−	−
2	73	M	23/30	+	AS	−	−		−	82	35	−	−	+	+	+	−
3	66	M	23/30	−	AS+AR	−	−		−	38	34	+	−	−	−	−	−
4	69	M	30/34	−	AR	−	+		+	71	43	−	−	−	−	−	−
**AF**																	
S/S1	49	M	23/23	−	MS	<1	−		+	60	54	−	−	−	−	−	−
S/S2	47	F	21/23	−	MS	6	−		−	52	52	−	−	−	−	−	−
S/S3	63	M	22/23	+	CABG	<1	−		+	48	41	+	+	−	+	+	−
S/S4	79	M	22/25	−	MR	<1	−		−	59	59	−	−	−	−	−	−
S/S5	51	M	23/24	−	MS+AR	4	−		−	61	61	−	+	−	−	−	−
S/S6	62	F	22/23	−	MS	4	−		−	66	50	+	+	−	−	−	−
L/L1	74	F	30/31	−	MS	7	−		−	70	74	−	+	−	+	−	−
L/L2	55	F	30/30	−	AS+MS	6	−		−	52	52	−	−	−	−	−	−
L/L3	48	F	30/30	−	MR	5	−		−	70	59	+	−	−	+	+	−
L/L4	55	F	30/36	−	AS+MS	6	−		−	74	56	−	+	−	+	+	−
L/L5	64	F	30/35	−	MS	3	−		−	68	56	−	−	−	+	+	−
L/L6	43	M	30/36	−	MR	10	−		−	67	81	−	+	−	−	+	−

### Effect of the GT-repeats on the HO-1 transcriptional activity in rapidly paced HL-1 myocytes

**Figure 2:**
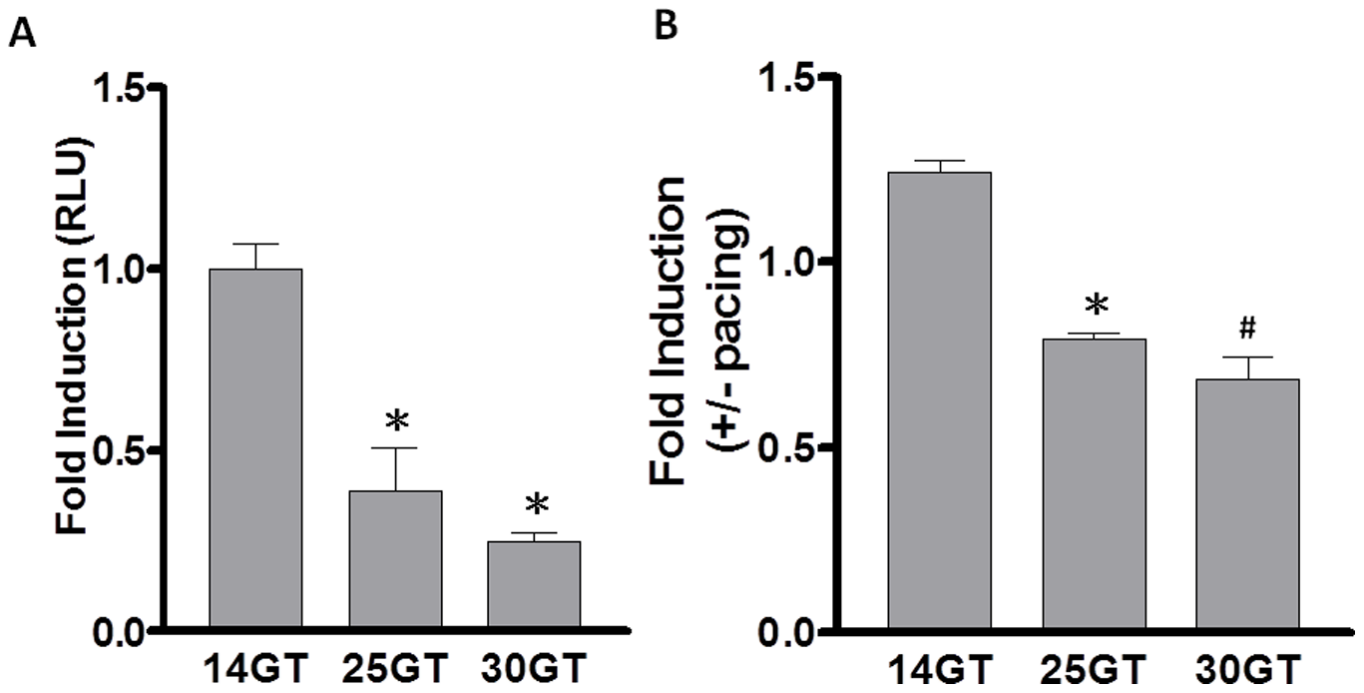
GT-repeats modulate the transcriptional activity of HO-1 gene and its responsiveness to rapid pacing. (**A**) HL-1 cells were transfected with plasmids containing various lengths of GT-repeats in the HO-1 promoter for 24 hours. The luciferase activity was assayed as described in Methods. (**B**) HL-1 cells were transfected with plasmids containing various lengths of GT-repeats in the HO-1 promoter for 24 hours and/or subsequently received tachypacing (4 Hz) for 2 hours. Each value (mean ± SE, [n = 4]) is expressed as a fold change of luciferase activity relative to the control condition. P<0.05; *, #: the different symbols represent the significant difference among groups.

Previous studies have demonstrated that rapid pacing of cultured HL-1 myocytes mimics the phenotype feature of tachycardia-induced atrial remodeling *in vivo* [20]–[23]. Accordingly, we utilized this atrial-derived system to evaluate the effect of tachypacing *in vitro*. To investigate whether the length of the GT-repeats affects the transcriptional regulation of the HO-1 promoter by tachypacing, human HO-1 promoter constructs containing different numbers of GT-repeats (14, 25, and 30) were generated and transfected into HL-1 myocytes. As shown in Figure 2A, a negative correlation between the promoter activity and the length of the GT-repeats was observed. Furthermore, there was a progressive decrease in pacing-induced HO-1 promoter activity when plasmids contained increasing numbers of the GT-repeats (Figure 2B). These results suggest that the length of the GT-repeats may modulate the transcriptional activity of HO-1 gene and its responsiveness to rapid pacing.

### Association of the GT-repeat length polymorphism with atrial structural remodeling in AF tissues

**Figure 3:**
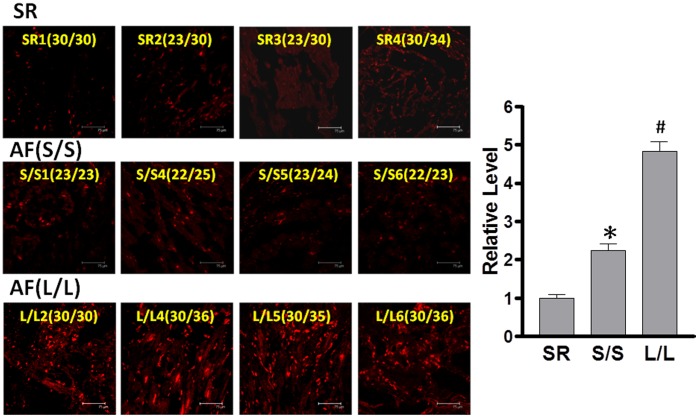
Association of the GT-repeat length polymorphism with oxidative stress in AF tissues. An identical paradigm was followed as described in Figure 1. Atrial tissues were stained with DHE to detect ROS generation as described in Methods. Representative confocal images show ROS production in the atria of 8 AF patients and 4 controls (sinus rhythm subjects [SR], left). Relative fluorescence density in the α-actin-expressing area was quantified (right). Data are expressed as mean ± SE. P<0.05; *, #: the different symbols represent the significant difference among groups.

**Figure 4:**
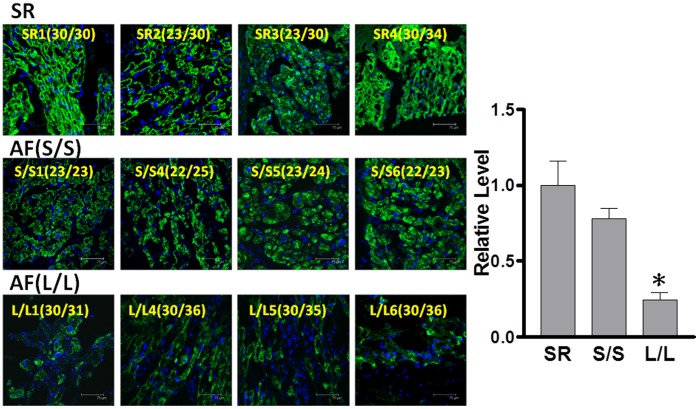
Association of the GT-repeat length polymorphism with myofibril degradation in AF tissues. Representative confocal images show myosin degradation in the atria of 8 AF patients and 4 controls (sinus rhythm subjects [SR], left). Relative intensity of MHC in the α-actin-expressing area was quantified (right). Data are expressed as mean ± SE. P<0.05; *: the different symbol represents the significant difference among groups.

**Figure 5:**
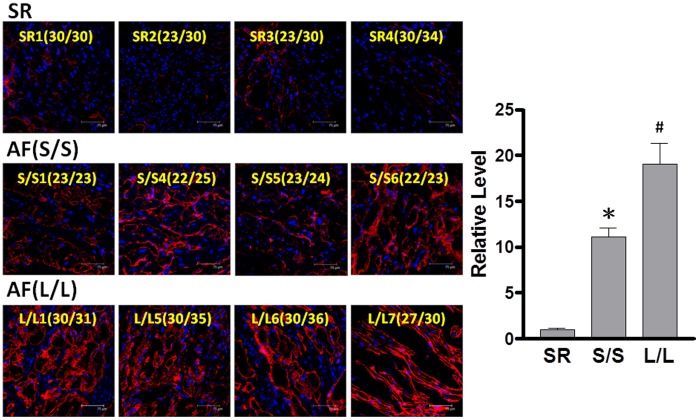
Association of the GT-repeat length polymorphism with fibrosis in AF tissues. Representative confocal images show fibrosis in the atria of 8 AF patients and 4 controls (sinus rhythm subjects [SR], left). Relative intensity of collagen I was quantified (right). Data are expressed as mean ± SE. P<0.05; *, #: the different symbol represents the significant difference among groups.

Our previous study showed that tachypacing promotes atrial structural remodeling, especially myofibril degradation in atrial myocytes, through increased oxidative stress [23]. To determine whether the GT-repeat polymorphisms affect AF-related oxidative stress and substrate remodeling, we further assessed the relationship between GT-length variants and oxidative stress, MHC degradation, as well as fibrosis in the atria of patients with AF. Consistently, we found that oxidative stress (indicated by increased ROS generation, Figure 3), myofibril degradation (indicated by decreased MHC expression, Figure 4), and fibrosis (indicated by increased collagen generation, Figure 5) were more severe in the atria of AF patients homozygous for longer alleles than those homozygous for shorter alleles and SR control. Taken together, these findings suggest that the GT-repeat length polymorphism may affect the HO-1 expression and the consequent atrial structural remodeling responses in AF tissues.

## Discussion

Electrical, contractile, and structural remodeling in the atrium constitute the main features of AF [1]. These morphological and electrophysiological alterations trigger a positive-feedback process to exhibit the self-perpetuating behavior of AF [1]. Tachycardia-induced oxidative stress has been recognized as an important mediator in promoting AF and its perpetuation [1]. Studies from our group and others have shown that oxidative stress mediates tachycardia-stimulated atrial remodeling [12]–[15], [23]. Therefore, the responsiveness of individual atrium to oxidative stress and the protective response by means of up-regulation of antioxidant genes may determine their vulnerability to AF. In this study, we have demonstrated HO-1 to be a prime candidate gene involved in these processes.

Our findings agree with a previous study showing that AF patients have higher HO-1 levels in their left atria than controls, especially in the posterior wall of the left atrium [25]. Our study provides further information that not only the location but also the genetic variation determines the HO-1 expression in the atria of AF patients. We also demonstrate that the abundance of HO-1 is accompanied by a lesser degree of atrial structural remodeling in AF patients. It is conceivable that the HO-1 expression may locally protect the atrium from AF-induced remodeling. Because antioxidant therapy, such as statin treatment, is found to prevent only early oxidative stress in AF patients [26], individuals may still develop AF if oxidative insult persists despite having a good (possibly a short GT-repeat) genetic background. Recently, Hu et al. investigated the effect of HO-1 microsatellite polymorphism on the outcome after catheter ablation in 205 Taiwanese with drug-refractory AF [27]. Interestingly, they found that shorter GT repeats (higher HO-1 activity and expression) were associated with a higher AF recurrence after catheter ablation, suggesting HO-1 may offer a protective effect against ablation-induced myocardial damage [27].

Our study also supports the notion that the long GT-repeats in the HO-1 promoter region may interfere with its gene transcription, and subsequently reduce protection against oxidative stress and AF. In this study, we found that tachypacing-induced HO-1 promoter activity inversely correlates with the length of the GT-repeats in the HO-1 promoter. Besides in our HL-1 atrial myocytes, the longer GT-repeats have also been found to reduce the HO-1 transcriptional activity in several other cell types, including Hep3B cells [6] and vascular smooth muscle cells [7]. It has been suggested that the longer GT-repeats may promote a change in DNA confirmation, which in turn negatively affects HO-1 transcriptional activity and then the response to oxidative stress [9]. However, we still could not rule out the possibility that the observed association in our study is through other functional polymorphisms in linkage disequilibrium with the GT-repeats polymorphism. Taken together, our findings suggest a contribution of genetic polymorphism and the consequent variation in antioxidant capacity in the pathogenesis of AF.

Some limitations exist in our data. First, our findings were obtained from only 1 sample with a modest size. The genetic association study also has suspect validity when Bonferroni correction is stringently applied for multiple tests. Replication in a second cohort with larger sample size would improve the strength of the analysis. Second, the cross-sectional nature of our study cannot identify the cause and effect relationships between HO-1 expression and AF-related atrial remodeling, nor can HO-1 promoter polymorphism predict the future occurrence of AF in a normal individual. Additionally, it has been reported that there is a region-specific HO-1 expression in the left atria of AF patients [25]. Ethical constraints prevent us from comparing the regional differences among patients with different genotypes. For the same reason, we could not obtain larger atrial samples, which was thus inadequate for further HO-1 protein quantification by western blot in addition to the DNA isolation and immunohistochemical (qualitative/semi-quantitative) analysis. Finally, the examined subjects were ethnically Chinese, and hence, caution should be exercised when extrapolating our results to other ethnic groups.

## Conclusion

In summary, we have demonstrated that the length polymorphism of the GT-repeats in the HO-1 promoter determines gene expression in atrial myocytes and is associated with the risk of AF and AF-related structural remodeling. These findings provide further evidence that genetic variation may influence the responsiveness to oxidative stress and the susceptibility to AF.

## Supporting Information

Figure S1 **Frequency distribution of GT-repeats in controls (n = 240) and AF patients (n = 200).**
(TIF)
