# Supplementary figures and images for: Microsatellite Polymorphism in the Heme Oxygenase-1 Gene Promoter and the Risk of Atrial Fibrillation in Taiwanese
Source: PLoS One. 2014 Sep 30;9(9):e108773. doi: 10.1371/journal.pone.0108773 (PMC4182563; doi:10.1371/journal.pone.0108773)

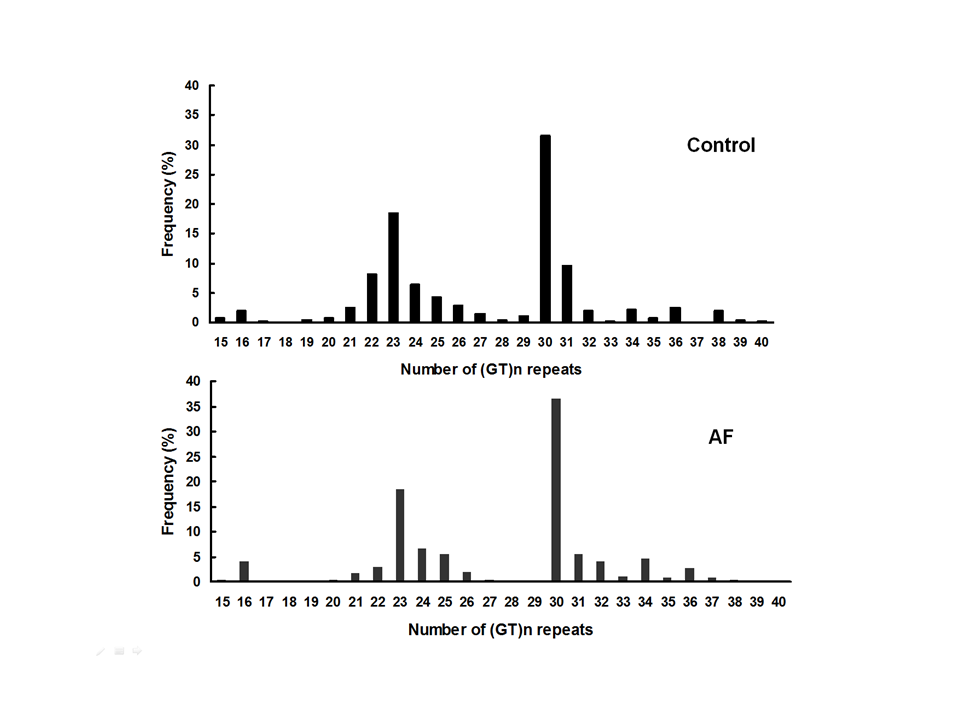

Supplement: Figure S1 — Frequency distribution of GT-repeats in controls (n = 240) and AF patients (n = 200). (TIF) [file pone.0108773.s001.tif]
